# Encapsulation of Orange Peel Oil in Biopolymeric Nanocomposites to Control Its Release under Different Conditions

**DOI:** 10.3390/foods12040831

**Published:** 2023-02-15

**Authors:** Sanaz Ghasemi, Elham Assadpour, Mohammad Saeed Kharazmi, Shima Jafarzadeh, Masoumeh Zargar, Seid Mahdi Jafari

**Affiliations:** 1 Department of Food Materials and Process Design Engineering, Gorgan University of Agricultural Sciences and Natural Resources, Gorgan 49189-43464, Iran; 2 Food Industry Research Co., Gorgan 49138-15739, Iran; 3 Food and Bio-Nanotech International Research Center (Fabiano), Gorgan University of Agricultural Sciences and Natural Resources, Gorgan 49138-15739, Iran; 4 Faculty of Medicine, University of California, Riverside, CA 92679, USA; 5 School of Engineering, Edith Cowan University, Joondalup, WA 6027, Australia; 6 Nutrition and Bromatology Group, Department of Analytical Chemistry and Food Science, Faculty of Science, Universidade de Vigo, E-32004 Ourense, Spain; 7 College of Food Science and Technology, Hebei Agricultural University, Baoding 071001, China

**Keywords:** limonene, biopolymeric nanocomposite, release kinetics, encapsulation

## Abstract

Orange peel oil (OPO) is one of the most common flavorings used in the food industry, but it is volatile under environmental conditions (the presence of light, oxygen, humidity, and high temperatures). Encapsulation by biopolymer nanocomposites is a suitable and novel strategy to improve the bioavailability and stability of OPO and its controlled release. In this study, we investigated the release profile of OPO from freeze-dried optimized nanocomposite powders as a function of pH (3, 7, 11) and temperature (30, 60, and 90 °C), and within a simulated salivary system. Finally, its release kinetics modelling was performed using experimental models. The encapsulation efficiency of OPO within the powders, along with the morphology and size of the particles, were also evaluated by an atomic force microscopy (AFM) analysis. The results showed that the encapsulation efficiency was in the range of 70–88%, and the nanoscale size of the particles was confirmed by AFM. The release profile showed that the lowest and the highest release rates were observed at the temperatures of 30 and 90 °C and in the pH values of 3 and 11, respectively, for all three samples. The Higuchi model provided the best model fitting of the experimental data for the OPO release of all the samples. In general, the OPO encapsulates prepared in this study showed promising characteristics for food flavoring applications. These results suggest that the encapsulation of OPO may be useful for controlling its flavor release under different conditions and during cooking.

## 1. Introduction

Flavors are very valuable compounds in the food and pharmaceutical industries. Orange peel oil (OPO), extracted from citrus peels as a liquid food flavor, is volatile and unstable in the presence of light, oxygen, humidity, and high temperatures. It is a mixture of several chemical ingredients, mainly consisting of *d*-limonene (>90%), which is the base sensory characteristic of OPO and is very unstable under processing and environmental conditions [1]. Encapsulation is an effective strategy to minimize these unfavorable effects and improve the bioavailability and stability of OPO. Encapsulation allows the protection of flavors from environmental degradation and controls their release during shelf-life and consumption [2,3].

Biopolymer nanoparticles (NPs) are used as efficient nanocarriers in food and pharmaceutical systems and can be applied for the encapsulation of valuable bioactive compounds and nutraceuticals, such as flavors, vitamins, carotenoids, omega-3 fatty acids, flavonoids, sterols, etc. Biopolymer NPs can be produced alone or through clustering and chain-linking of a biopolymer (protein or polysaccharide), or by controlling the binding and complexing of protein molecules and polysaccharides [4]. Polysaccharide–protein NPs are applied for encapsulation more widely than pure and individual biopolymer NPs. The protein–polysaccharide composite is an effective carrier for the encapsulation of volatile substances under optimal conditions (in terms of protein: polysaccharide ratio, concentration, ionic strength, pH, and temperature). In fact, this complex is formed through covalent and/or electrostatic bonds [5,6]. The complexes can be produced in nano sizes by high-energy densities such as ultrasonication, which causes higher surface-to-volume ratios and can protect the encapsulated ingredients from degradation more efficiently [7,8]. Whey protein concentrate (WPC) is a combination of different globular proteins containing β-lactoglobulin, being the most dominant, followed by α-lactalbumin, which is used extensively in the production of protein–polysaccharide complexes [9]. On the other hand, pectin is one of the most popular hetero-polysaccharides obtained from natural sources, such as citrus fruits, apples, and sugar beetroots, with the main chain of galacturonic acid units and methanol partially esterified. Low-methoxyl pectin (LMP) is an anionic polysaccharide prepared from the de-esterification of high-methoxyl pectin [10].

Mathematical modeling of the release behavior of bioactives provides beneficial information related to the chemical processes and mass transfer of bioactives from encapsulated ingredients. It also shows how different factors such as morphology affect the release rate. Therefore, it is possible to predict the kinematic release with minimal experimental data, and then the optimized conditions for the release profile of encapsulated bioactives can be determined [9,11]. There have been extensive studies on the release kinetics of bioactives and nutraceuticals from encapsulated environments, and the modeling of release behavior. For instance, Fathi et al. [12] investigated the release kinetics of hesperidin from NPs of solid lipids in simulated gastrointestinal conditions using the zero-order, first-order, Higuchi, and Pepas mathematical models. Their results showed that the Pepas and the zero-order models were the best and the worst models, respectively, for almost all the samples to describe the release of hesperidin, based on the correlation coefficient [12]. Alvarado, et al. [13] evaluated ferrous bisglycinate release kinetics from W_1_/O/W_2_ double emulsions stabilized by a complex of WPC with the polysaccharide of gum arabic (GA), mesquite gum (MG), or LMP, in the pH range of 1.5–5, and fitted the data to the models of zero-order, first-order, Hixson–Crowell, and Higuchi. Their results showed that the W_1_/O/W_2_ multiple emulsions stabilized with WPC:MG (5% *w/w* total biopolymers concentration) provided smaller droplet sizes (2.05 μm), better protection against ferrous bisglycinate oxidation (29.75% Fe^3+^), and a slower rate of ferrous bisglycinate release from W_1_ to W_2_. In addition, their results indicated that the thickness of the protein (i.e., polysaccharide complex adsorbed around the multiple emulsion oil droplets, which were determined by measuring the z-average diameter of the complexes) was the key factor affecting the release kinetics of the ferrous bisglycinate from the inner aqueous phase of the multiple emulsions to the outer aqueous phase. The thicker the protein–polysaccharide complex interfacial membrane in the multiple emulsions—the slower the release kinetics [13].

Assadpour et al. [9] evaluated the release kinetics of folic acid-loaded nano double W_1_/O/W_2_ emulsion. First, they prepared folic acid-loaded W_1_/O nano-emulsions, and then re-emulsified them into an aqueous phase (W_2_) containing a single layer of WPC, or a double layer of WPC–pectin complex, to form W_1_/O/W_2_ emulsions. They evaluated the release profile of the folic acid in aqueous solutions with predetermined pH values (4, 7, 11) at 37 °C. The solutions were stirred at 750 rpm for different time intervals from 5 to 60 min. The release kinetics of the folic acid was calculated using the zero-order, first-order, Higuchi, and Hixson–Crowell experimental models. Their results showed that folic acid nano-capsules made with Span surfactant had the lowest and highest release rates at the pH values of 4 and 11, respectively. The single-layer WPC-encapsulated powders had the best model-fitting for the release of folic acid data with the highest R^2^.

In the present work, OPO-loaded WPC–pectin nanocomposites were produced, and the optimum nanocomposite was selected based on different levels of WPC (4, 6, 8 g), pectin (0.5, 0.75, 1 g), and pH (3, 6, and 9) through response surface methodology (RSM) and conducting analyses such as color, viscosity, and stability on the nanocomposites. The main goal was to investigate the release profile of OPO from freeze-dried optimized powder particles as a function of pH (3, 7, and 11) and temperature (30, 60, and 90 °C). It was expected that acidic pH values and lower temperatures could control the release of OPO from the complexes. Then, its release kinetics modeling was performed using the experimental models of zero-order, first-order, Higuchi, and Hixson–Crowell to find the best-fitting model. The final goal was to evaluate the release of OPO from the powders in a salivary simulated system and to find the best formulation for applying encapsulated OPO in food products, such as fruit juices.

## 2. Materials and Methods

### 2.1. Materials

OPO (including >96% *d*-limonene) and Tween 80, as the emulsifying agent, were purchased from Ramsar Citrus Concentrate Co. (Ramsar Citrus Concentrate Co., Ramsar, Iran) and Sigma-Aldrich Co. (St. Louis, MO, USA), respectively. Citrus LMP, WPC (80% *w/w* protein, 3.5% ash, 6% moisture, and 0.45 g/cm^3^ bulk density), and maltodextrin (MD; DE = 16–20) were purchased from Sigma-Aldrich Co. (USA), Arla (Denmark), and Qinhuangdao (China), respectively. Double distilled water was used for the preparation of all the solutions. The enzyme of α-amylase (from a porcine pancreas), DFP-treated, Type I-A, saline suspension, ≥1000 units/mg protein (E1%/280) was purchased from Sigma-Aldrich Co (USA). All the other chemicals used in this study were of an analytical grade.

### 2.2. Encapsulation Process of Orange Peel Oil in Whey Protein–Pectin Complexes

**Figure 1:**
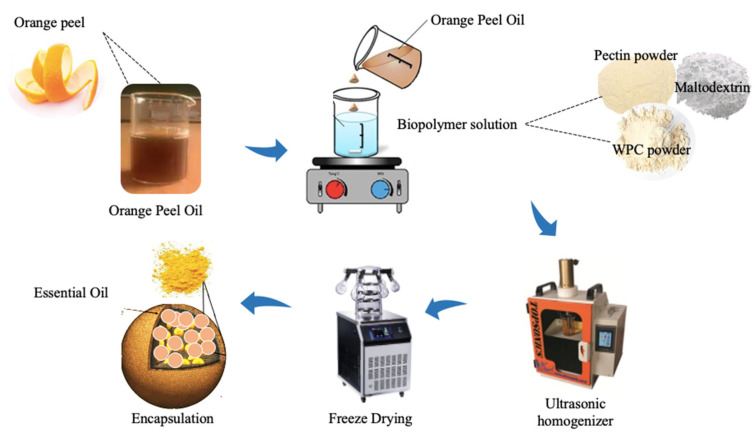
Encapsulation process of orange peel oil.

According to Figure 1, for preparing 100 mL pectin solutions, pectin powder (0.5, 0.75, 1 g) was dissolved in deionized water (70 °C). Then, different WPC aqueous solutions (100 mL) were prepared by dispersing the required WPC powder content (4, 6, 8 g) in deionized water. In addition, maltodextrin at a ratio of 50% *w/v* (at constant concentration) was prepared and added into the mixed biopolymer solutions as a filler. These solutions were stirred for at least 30 min on a magnetic stirrer (IKA, Germany) and stored overnight at room temperature to complete the hydration of the biopolymers [2].

The prepared solutions of pectin, WPC, and maltodextrin were mixed together in the same volumes and stirred for 30 min on a magnetic stirrer. Then, Tween 80 was added to the solutions at the ratio of 10% of the total solids and mixed to dissolve entirely. After that, OPO was added into each solution at the ratio of 20% of the total solids (a constant ratio that was obtained in pre-tests) gradually under homogenization by an ultrasonic homogenizer (Iranian Ultrasonic Technology Company, 20 kHz, 400 W, 12 mm probe diameter) at a power of 350 W and a temperature of 25 °C for 10 min. The pH of the solutions was adjusted to predetermined pH values (3, 6, and 9) using HCl and NaOH (0.1 and 1 N), and the complexes were formed [2].

The optimum nanocomposite was selected through the central composite design pattern of the response surface methodology (Design Expert Software, State-Ease Co., version 10). This was based on the viscosity, color (L*), and stability analysis, which was measured by a viscometer (LVDV Pro II, Brookfield Engineering, spindle S00, USA), Image J software (image analysis), and accelerated centrifugation (Sigma Co., 3K30 model) at 20,000× *g* and 25 °C for 30 min, respectively [7,14,15]. The optimum nanocomposite (optimized ratio of WPC and pectin) was formulated in three different pH values (3, 6, and 9) and converted into powdered forms by freeze-drying (FDB5503 Freeze-dryer; Operon, Gyeonggi-do, Republic of Korea); the samples were named NC3, NC6, and NC9, respectively. The freeze-dried samples were ground into a suitable powder by a pestle and mortar [7].

### 2.3. Encapsulation Efficiency of Orange Peel Oil

It is necessary to analyze the encapsulated nanocomposite powders to determine the total content and surface content (non-encapsulated) of OPO in them. Accordingly, 0.5 g of each powder was dispersed in 20 mL hexane. After 2 min, the solution was filtered using a Whatman paper no. 41. The filtered solution was then analyzed using a UV–Vis spectrophotometer (T80, PG Instruments Ltd., UK) at 231 nm wavelength to identify the OPO content on the surface of the powders (NC3, NC6, NC9) (i.e., non-encapsulated fraction) (Matias et al., 2014). To identify the total content of OPO, 0.5 g of the powders were mixed in 20 mL distilled water (pH = 6–7) and vortexed (high power) until completely dissolved. The prepared solution was then mixed with 20 mL hexane and vortexed for a further 5 min. After that, the solution was centrifuged at 3500× rpm for 20 min, and the supernatant was used to determine the total content of OPO using the UV–Vis spectrophotometer at the wavelength of 231 nm. The encapsulation efficiency (EE%) of the OPO was calculated using Equation (1) [16,17]. The result of EE% was applied in estimating the amount of OPO release:

(1) $$\;\mathrm{EE}\%=\left(\mathrm{Total}\;\mathrm{amount}\;\mathrm{of}\;\mathrm{loaded}\;\mathrm{oil}-\mathrm{surface}\;\mathrm{content}\;\mathrm{of}\;\mathrm{oil}\right)/\left(\mathrm{Total}\;\mathrm{amount}\;\mathrm{of}\;\mathrm{loaded}\;\mathrm{oil}\right)\times 100\;$$

### 2.4. Atomic Force Microscopy Analysis

An atomic force microscope (AFM) (DualScopeTM DS95-50, DME, Denmark) was used for the morphological characterization and analyzing the particle size and size distribution of nanocomposites of the WPC–pectin. A drop of diluted nanocomposite suspension (0.05 mg/mL) was applied on a clean glass surface and dried at room temperature for the preparation of the AFM samples. Then, the AFM images for NC3, NC6, and NC9 were analyzed [18].

### 2.5. Release Profile Analysis of Orange Peel Oil

To determine the release profile of OPO from the pectin–WPC nanocomposites (NC3, NC6, and NC9), 0.5 g of encapsulated OPO powder was dispersed in 50 mL aqueous solutions with adjusted pH values of 3, 7, and 11. These suspensions were then stirred on a magnetic stirrer (750 rpm) at different temperatures (30, 60, 90 °C) for various time intervals from 30 to 1800 s. After that, a 1 mL sample at each time point was taken out during the stirring and filtered by Whatman paper No. 41. Finally, 0.3 mL from each taken sample was mixed with 3 mL hexane, vortexed for 2 min, and then centrifuged (3k30, Sigma, USA) at 3500× rpm for 15 min. The content of OPO was then determined spectrophotometrically (T80, PG Instruments Ltd., England) at 231 nm [19]. The amount of encapsulated OPO was determined through EE %; so, by estimation of the amount of released OPO, the percentage of the released content can be determined. The release kinetics of OPO were calculated using the following model Equations (2)–(5) [20]:

(2) $$\mathrm{Zero-order}:\;Q_{t}=Q_{0}+K_{0}\;t\;$$

(3) $$\mathrm{First-order}:\;Log\;C_{t}=Log\;C_{0}-K_{1}t/2.303$$

(4) $$\mathrm{Higuchi}:\;{\left[Q\right]}_{t}={\left[k\right]}_{h\times t/2}\;(\mathrm{Higuchi},\;1961)$$

(5) $$\mathrm{Hixson-Crowell}:\;[C_{0}]{\;}^{\frac{1}{3}}-\left[n\left(C_{t}\right)\right]{\;}^{\frac{1}{3}}=KHC_{t}$$

where *k* is the model constant, *Q_t_* is the released concentration of OPO at any time, *t* is time, *Q*_0_ is the initial concentration of OPO in the solutions (usually *Q*_0_ = 0), *C*_t_ is the remained concentration of OPO within the capsules after time *t*, and *C*_0_ is the initial concentration of OPO within the capsules.

### 2.6. Determination of the Release Profile in Simulated Salivary Conditions

The notable issue for the WPC–pectin complexes containing OPO is whether α-amylase can hydrolyze the complex or not, because the salivary system contains α-amylase and other minerals and enzymes. Therefore, it was expected that the simulated salivary fluid would be able to hydrolyze maltodextrin within the structure of complexes during chewing. This results in the release of the OPO, which is favorable in terms of sensory properties when this carrier system is going to be applied in food formulations. This part of the study was designed to evaluate the hydrolysis of WPC–pectin complexes containing OPO and the amount of release in a simulated salivary condition. The release profile of OPO in salivary conditions was determined by incubating OPO-loaded nanocomposites in simulated salivary conditions. For this purpose, 200 mg of the WPC–pectin nanocomposite powder was incubated in the simulated salivary fluid (SSF) (temperature of 37 °C, pH = 6.8 and enzymatic activity of 100 μg/mL) for 2 min [21,22]. The SSF was an aqueous solution with a pH of around 6.8 (pH was adjusted by potassium hydroxide), which contained 10.0 g/L sodium carboxymethyl cellulose, 2 g/L methyl-p-hydroxy benzoate as a preservative, 0.29 mmol/L magnesium chloride, 8.38 mmol/L potassium chloride, 4.62 mmol/L dikalium hydrogen phosphate, 1.13 mmol/L calcium chloride, 0.022 mg/mL fluoride, and 2.40 mmol/L kalium dihydrogen phosphate to simulate human salivary conditions [23]. Sodium carboxymethyl cellulose increases the viscosity of the artificial salivary fluid in order to mimic the viscous flow of natural salivary fluid and other materials, and provide the inorganic components in natural salivation. Another important ingredient in SSF is amylase. The quantity of applied α-amylase was 100 units/mL to simulate the average activity while chewing [24]. Incubation was performed in a 50 mL beaker filled with 20 mL SSF at 37 °C under continuous stirring (100 rpm). The samples were taken after 1 min and 2 min incubation, and the extent of released OPO from the nanocarriers in the SSF was measured by spectroscopic absorbance at the wavelength of 231 nm [21,24].

### 2.7. Statistical Analysis

In the first step of the study, EE% was estimated, and the size and morphology were evaluated for the NC3, NC6, and NC9 samples by AFM analysis. Then, the influence of various pH values (3, 7, 11) and different temperatures (30, 60, 90 °C) on the release profile of the NC3, NC6, and NC9 samples was investigated by factorial experiments through SAS Software (version 9) in two replicates. Further, in the simulated salivary conditions, the release rate of the three powders was evaluated. The graphs were drawn by Minitab software (version 16) and Excel (version 2010). MATLAB software (version 2012) was applied for fitting the release data with the kinetic models. The best fitting of the models with the release data was determined, based on a high correlation coefficient.

## 3. Results and Discussion

### 3.1. Results of Encapsulation Efficiency for Powders

**Figure 2:**
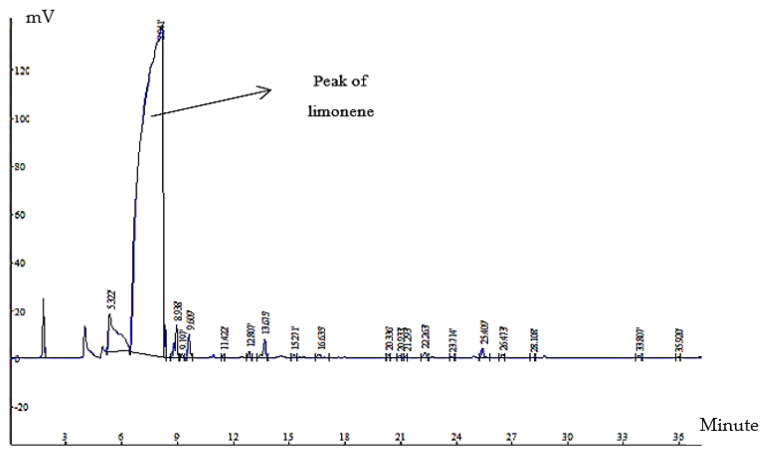
Chromatogram related to gas chromatography of OPO.

According to the gas chromatography performed on OPO (Figure 2), which showed that limonene is the main ingredient in OPO, and the review of previous studies that used the wavelength of 231 nm for limonene as the wavelength that has the most absorption, we used a wavelength of 231 nm to check the amount of limonene inside the capsules.

The EE% was calculated using Equation (1). It was revealed that the EE% of the NC3, NC6, and NC9 powders was 88%, 84%, and 70%, respectively. The maximum EE% value was achieved in pH = 3, as the complex coacervate was formed with a higher and stronger entrapment of OPO within the nanocomposite particles. It was revealed that the increase of pH from 6 to 9 decreased the EE% of the OPO within the produced nanocomposites, which can be due to the weaker complex formation at higher pH values [9,16,23]. At higher pH values (above the isoelectric point of proteins), the amount of OH^−^ groups on the surface of amino acids is increased more than the H^+^ groups, so the negative charges on the protein will be dominated; the pectin is also negatively charged, due to the presence of carboxyl groups on its surface. As a result, stronger repulsive forces are generated between the WPC and pectin than the electrostatic interactions between the anionic carboxyl groups of pectin and positively charged groups of WPC, resulting in weaker complexes being formed at higher pH values [9,16,23].

### 3.2. Evaluating the Morphology and Size of Nanocomposites

**Figure 3:**
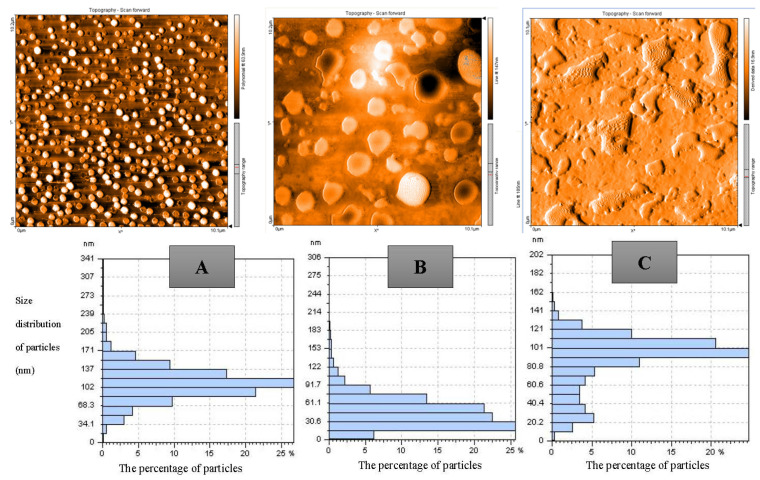
AFM images (3D) and particle size distribution obtained from WPC–pectin complexes in three powders: (**A**) NC3, (**B**) NC6, and (**C**) NC9.

The morphological characteristics, particle size, and particle size distribution of WPC–pectin complexes (NC3, NC6, NC9) were determined using AFM. The AFM images confirmed the nano-size structure and spheroid-like appearance of the WPC–pectin complexes on a 10.1 × 1.2 µm scale (Figure 3). The images in terms of particle size showed that the maximum amounts of WPC–pectin nanocomposites in NC3, NC6, and NC9 samples had an average size of 110, 20, and 90 nm with a size distribution of 20–210, 0–170, and 10–160 nm, respectively. According to the size distribution data in the NC9, particles of different sizes are seen because the complex was not well-formed. Still, the size of the particles in the NC6 and NC3 was in the range of nano, and more concentrated around, a specific size. The comparison between the AFM images of the samples showed that smaller and larger WPC–pectin NPs were observed for the NC6 (Figure 3B) and NC3 samples, respectively (Figure 3A). The nanocomposite particles of the NC3 were more spherical than the particles in the NC6 and NC9, which may be due to the complex coacervation and aggregation of the WPC–pectin complexes. In the NC9 powder, both biopolymers had negative charges, and the positive charges on the groups of WPC were also lower. Therefore, the repulsion between these two biopolymers was higher, and the detachment of pectin from the WPC surfaces occurred, resulting in bigger particle sizes (Figure 3) [7,18,25].

### 3.3. Influence of Different pH Values on the Release of Orange Peel Oil

**Figure 4:**
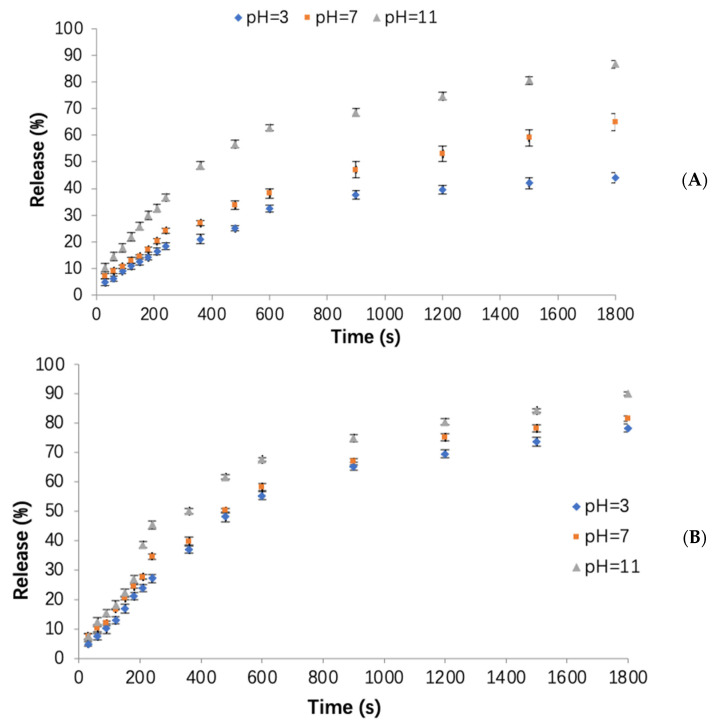

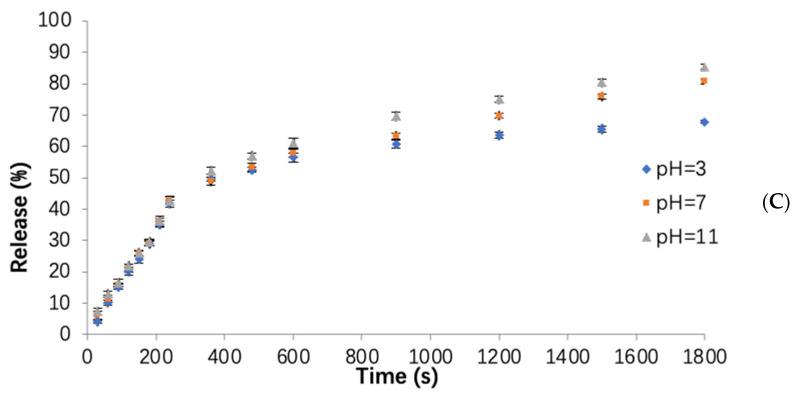
The release of orange peel oil from powders at 60 °C and different pH values (3, 7, 11): (**A**) NC3, (**B**) NC6, (**C**) NC9.

The release profile analysis of the OPO from the three types of freeze-dried WPC–pectin nanocarrier powders (NC3, NC6, NC9) is shown in Figure 4. In all three powders, the highest release rate was observed in the first 0–240 s. It is most probably due to the structural changes in the first 0–240 s, due to swelling and the structural re-organization of the nanocomplexes. This would mean a concentration- and/or time-dependent diffusion phenomenon, which can be described by case II diffusion models [26]. This release behavior suggests a dual diffusion process; i.e., probably two values of diffusivity could be calculated, one at the very first stages when swelling occurred, and another much greater after the structural changes. In order to simplify the calculations, the release rate of the OPO in each time interval was determined by calculating the slope of the relevant lines. In the time interval of 15–30 min, it followed faster kinetics, depending on the pH. As can be seen in Figure 4, at higher pH values (pH = 11), the release of the OPO was increased, and it had maximum values in alkaline conditions, which could be due to disintegration and better solubility of the complexes, which is caused by better solubility of WPC and faster decomposition of pectin in alkaline conditions, similar to the results of Assadpour et al. [7]. On the other hand, the solubility of WPC at around its isoelectric point (pI ≈ 4) reached the lowest values [27], so that, under acidic conditions (pH = 3), nonencapsulated powders precipitate and agglomerate. Therefore, it is difficult for these powders to dissolve and release their bioactive content completely. Generally, the release rates of the OPO in alkaline (pH = 11) and acidic (pH = 3) conditions were higher and lower, respectively, which could be desirable as it is important for nutraceutical compounds to tolerate and pass the acidic conditions of the stomach, reach the intestine, and release their cargo in alkaline conditions for their absorption in cells [28].

The release rates of the OPO from the NC3 powder at three pH values (3, 7, 11) were the lowest values, as shown in Figure 4A. The release rates for this powder at pH = 3, 7, and 11 were obtained as 0.06, 0.08, and 0.12, sequentially. The rates of released OPO at pH = 3, 7, and 11 were 18, 24, and 36%, respectively, after 240 s. In the following time interval (240–600 s), the release rate of the OPO was reduced in all the pH ranges, and the release rates for pH = 3, 7, and 11 were 0.05, 0.07, and 0.08, respectively. Finally, in the time of 600–1800 s, it was decreased again, and, for the pH = 3, 7, and 11, it was equal to 0.01, 0.01, and 0.02, respectively, which were almost the same for all the studied pH ranges. In fact, the NC3 powder had the lowest and highest release rates at pH = 3 and pH = 11, respectively. In the NC6 powder (Figure 4B), the release rate of OPO at pH = 3, 7, and 11 was observed about 0.1, 0.13, and 0.16 at 0–240 s, sequentially. After 240 s, the NC6 released 27, 35, and 45% of its total OPO content at pH = 3, 7, and 11, respectively; therefore, the release of the NC6 sample was faster and greater than the NC3 powder in the time interval of 0–240 s, because the formed WPC–pectin complexes in the NC6 powder were weaker than their NC3 counterparts. For the NC9 powder (Figure 4C), the release rate of OPO for all the pH values was 0.17 between 0 and 240 s. It was 0.03, 0.04, and 0.04, for pH = 3, 7, and 11 at 240–600 s, respectively, and it was 0.01, 0.02, and 0.02 at 600–1800 s, sequentially. Generally, the rate and the amount of release over time was higher for the NC9 powder than the NC3 or NC6, due to the weaker formed complexes in the NC9 powders [7]. During the first 240 s, the NC9 released 40, 41, and 42% of its total OPO at pH = 3, 7, and 11, respectively.

By comparing these three types of prepared nanocarriers, it was found that the rate of release in the first (0–240 s) and second (240–600 s) phases for the NC3 sample was <NC6 and NC9 powders, because the formed WPC–pectin complexes in the NC3 delivery system were stronger and more protective, so they could entrap and protect the OPO more effectively. In addition, the release rate in the NC6 powder was <NC9 powder, due to the formation of stronger complexes in the NC6 sample. A statistical analysis also showed that the influence of pH on the release rate in all three powders was significant (*p* < 0.05). When OPO is used as an encapsulated nutraceutical compound in food formulations, its protection is very important to reach the intestine. Accordingly, the NC3 powder proved to be the optimum nanocarrier among the three to be used as a functional food ingredient, due to its higher tolerance in the acidic conditions of the stomach with a lower release rate, while the OPO can be released in the alkaline conditions of the intestine at the highest rate [29,30].

### 3.4. Influence of Different Temperatures on the Release of Orange Peel Oil

**Figure 5:**
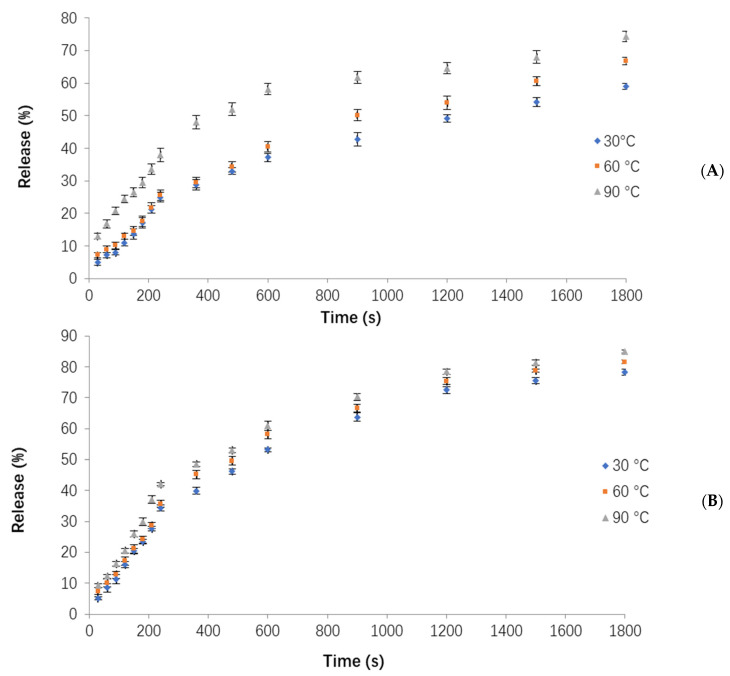

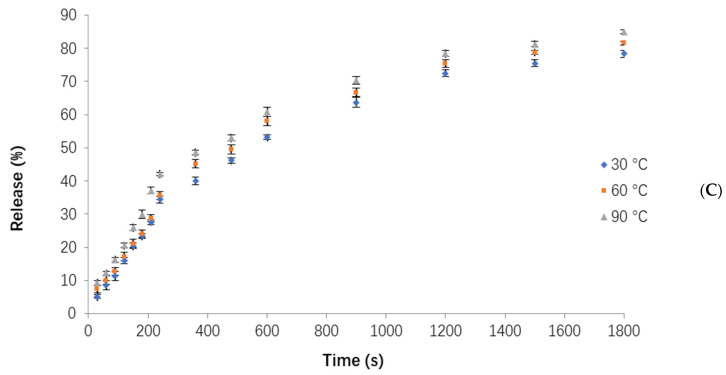
The release of orange peel oil from powders at pH = 7 and different temperatures (30, 60, 90 °C): (**A**) NC3, (**B**) NC6, (**C**) NC9.

Figure 5 shows the release profile of OPO from the powder particles of three nanocarriers made with complexes of WPC–pectin, prepared at pH = 7, and three temperatures (30, 60, and 90 °C). Our results revealed that, at higher temperatures, the release rate was increased, so the maximum release was observed at 90 °C, but generally speaking, except for the NC3 (Figure 5A) sample, the release rates were not much different at various temperatures. That can be due to the stronger formation of the complexes of NC3 powder, which could further retain the OPO. Therefore, the release rate of NC3 was lower at first and increased when the temperature was raised, resulting from the degradation of the walls of the capsules at higher temperatures. The release of OPO from the NC6 (Figure 5B) and NC9 (Figure 5C) samples was high at the initial temperature but did not change much as the temperature increased. That can be attributed to the weaker WPC–pectin complexes formed in these two powders than those in the NC3 powder. Our findings agreed with the results of Assadpour et al. [7].

The influence of temperature on the release can be related to the faster dissolution of the capsule-wall construction at the higher temperatures. The NC3 powder (Figure 5A) had a release slope of 0.09, 0.09, and 0.11 at 30, 60, and 90 °C, respectively, in the time interval of 0–240 s, and almost 25, 26, and 38% of the total OPO was released in these conditions. That indicates a higher release rate at 90 °C. In the next time interval of 240–600 s, the release rate for this powder was lowered at all the temperatures, and the slope of the release in all the temperatures was equal to 0.05. In the final studied time interval (600–1800 s), the release slope was decreased further, compared to the previous time, and the release slope was 0.02, 0.2, and 0.01 at 30, 60, and 90 °C, respectively. For the NC6 powder (Figure 5B), the influence of temperature was lower, and the release rate was higher at all three temperatures compared to the NC3 powder. The NC6 powder released 34, 35, and 42% of its total OPO in the time of 0–240 s. These results showed that, at 90 °C, the release rate and the overall release percentage were higher than the other temperatures. In the time interval of 240–600 s, the release slope for all the temperatures was 0.05 and decreased less than the previous time. In the next time interval, of 600–1800 s, it was 0.01, 0.20, and 0.20 at 30, 60, and 90 °C, sequentially. The NC9 powder (Figure 5C) had a released content of 38, 42, and 44% from its total OPO, with a slope of 0.15, 0.17, and 0.17 at 30, 60, and 90 °C, respectively, in the time interval of 0–240 s. The release slope at all the temperatures for this sample was the same, and equal to 0.03 and 0.02 in the times of 240–600 s and 600–1800 s, sequentially. Therefore, as a general result, the release rate in the NC9 powder was approximately the same in all the temperatures.

It was found that the release rate of the NC3 powder in the time interval of 0–240 s was lower than the other two samples. That can be due to the stronger WPC–pectin complexes formed for the NC3, which could protect the OPO more effectively, resulting in its lowest release rate. The NC6 and NC9 powders had almost the same release rates and different release contents. The highest release content was observed in the NC9 powder at 90 °C. That can be attributed to the weaker complexes formed in the NC9. Further, the temperature of 90 °C resulted in the disintegration of the formed complexes and the increased release of the OPO. The statistical analysis results also revealed that the influence of temperature on the release rate in all three powders was significant (*p* < 0.05). For targeted delivery of OPO as a nutraceutical into the body, it is important to maintain a temperature of about 30 °C (close to our body temperature) and have an appropriate release rate at this temperature. On the other hand, the high temperatures of thermal processes and the storage temperatures should have minimum effects on the loss and the release of OPO during processing. Hence, the NC6 nanocarrier powder with the minimum effect of temperature on its release rate would be the optimal sample for these conditions [29,30].

### 3.5. Modeling of the Orange Peel Oil Release Profile in Different Conditions

**Figure 6:**
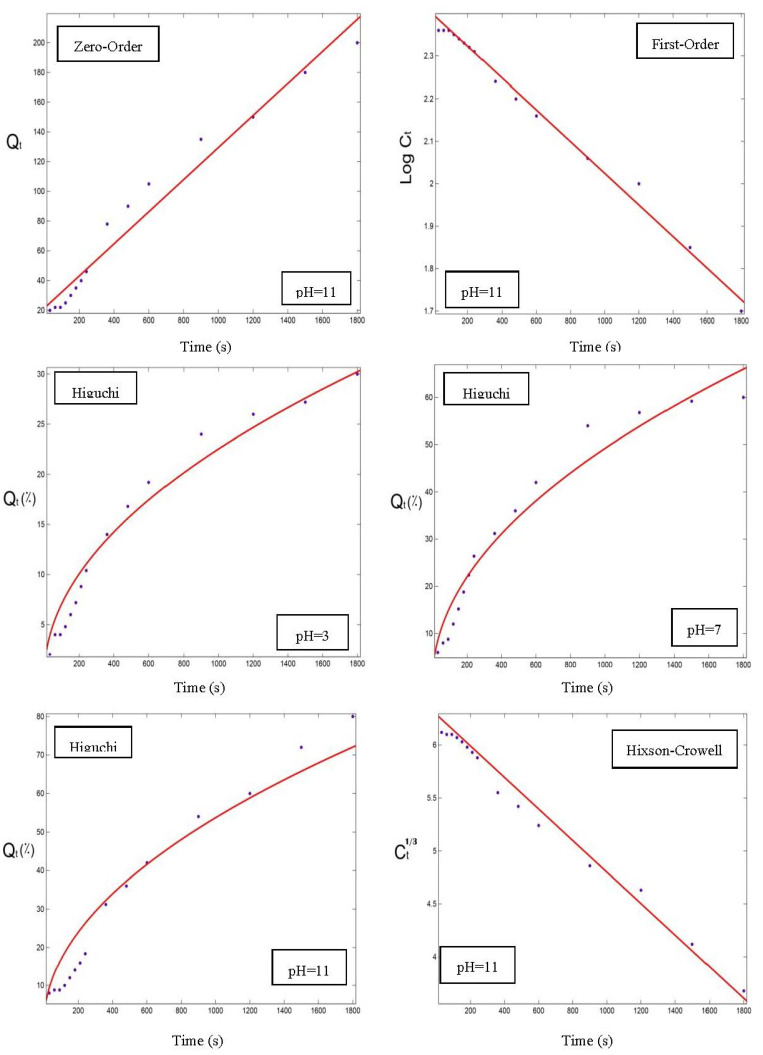
Release profiles (dots) and regression modeling (red lines) of orange peel oil dissolution from nano-delivery systems of pectin–whey protein powder particles (NC3) at 30 °C and different pH values.

**Figure 7:**
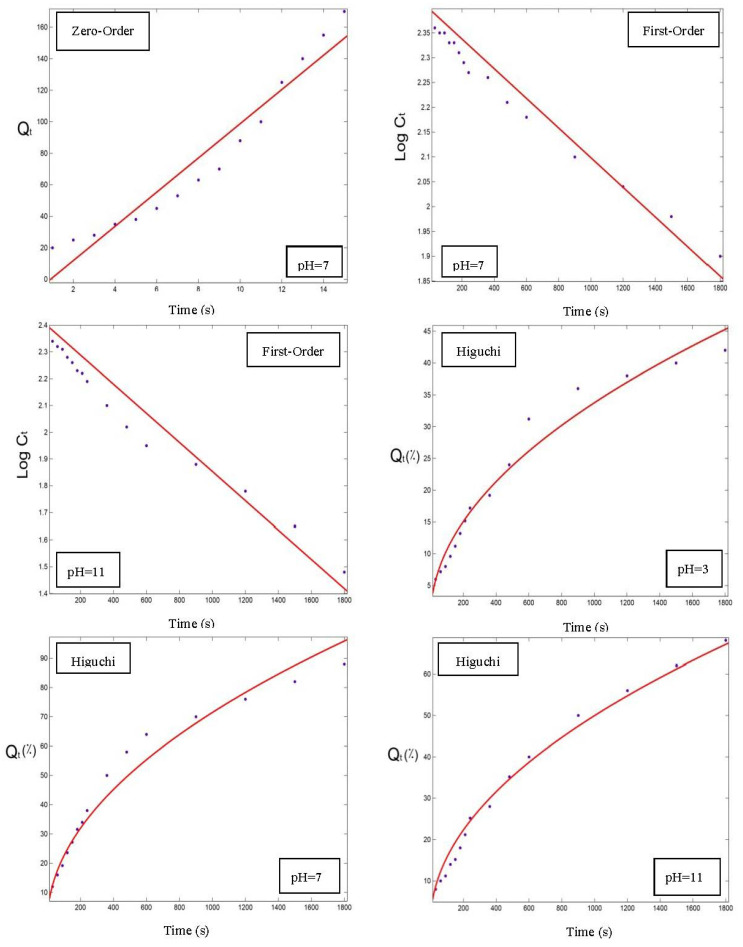
Release profiles (dots) and regression modeling (red lines) of orange peel oil dissolution from nano-delivery system of pectin–whey protein powder particles (NC3) at 60 °C and different pH values.

**Figure 8:**
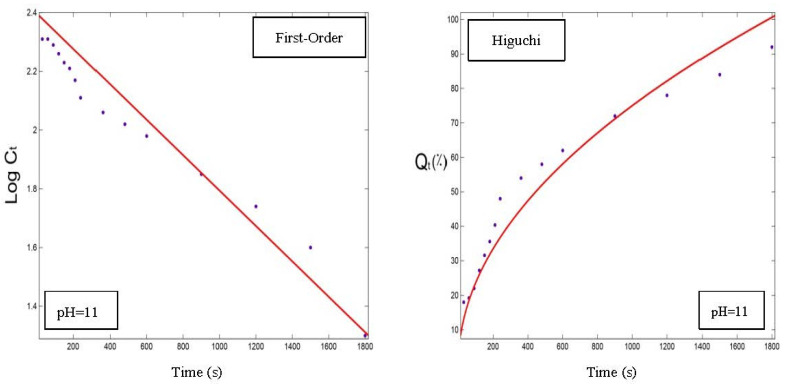
Release profiles (dots) and regression modeling (red lines) of orange peel oil dissolution from nano-delivery system of pectin–whey protein powder particles (NC3) at 90 °C.

In order to choose the best model for fitting the release data of OPO from the NC3, NC6, and NC9 powders, the parameters of each model were determined using the zero-order, first-order, Higuchi and Hixson–Crowell models. As an example, the OPO release rates of the NC3 powder are listed in Table 1, along with its corresponding correlation coefficients. The release profile in the two other powders was similar to the NC3 sample. The best release modeling was achieved in alkaline conditions (Table 1 and Figure 6, Figure 7 and Figure 8), based on the obtained correlation coefficients for all the samples, which was similar to the results of Assadpour et al., [7]. In addition, the results of the release modeling showed that the Higuchi and Hixson–Crowell models were the best and worst models, respectively, in terms of fitting the experimental release data for all three powders containing OPO.

As shown in Table 1, when the temperature was constant at 60 °C, the *k*-index for the Higuchi model at pH = 3, 7, and 11 was equal to 1.07, 1.58, and 2.26, respectively; and when the pH was constant at 7, the *k*-index was equal to 1.56, 1.58, and 2.06 at 30, 60, and 90 °C, respectively. Therefore, by increasing the pH and temperature, the complexes dissolved faster, resulting in higher release rates (k-index), and this trend had already been observed in the two other powders for the release profile. The remaining evaluated models showed the same trend and similar parameters. In other words, in a constant temperature (60 °C), the release rate at pH = 11 was 1.5 times >pH = 7 and 2 times >pH = 3. The reason could be that, in acidic conditions, encapsulated powder particles form precipitates and aggregate out of the solution, which makes it very difficult for the internal encapsulated OPO to release out of the capsules. Also, in a constant pH = 7, the release rate at 90 °C was almost 2 times >60 °C and about 1.3 times >30 °C. The results are consistent with the literature [7,31].

The fitted model diagrams for the release data of the NC3 powder with a correlation coefficient >0.9 are shown in Figure 6, Figure 7 and Figure 8, and the Higuchi model was determined as the best fitted model to the release data.

### 3.6. The Release of Orange Peel Oil in Simulated Salivary Conditions

The content of released OPO from the total core material in all three types of powders was determined, as shown in Table 2.

Our results revealed that, when the α-amylase was not present in the aqueous medium, the release of OPO was much lower, while, in the presence of α-amylase, the OPO was released at a higher rate. It was found that after 2 min, the NC3, NC6, and NC9 nanocomposite powders had a release of 36, 49, and 56% of the total OPO, respectively. According to the release data, it was obvious that the maltodextrin could be able to form a film as a bed for the nanocomposites and entrap some OPO. Hence, by adding the α-amylase into the solution and hydrolyzing the maltodextrin, more OPO was released. As shown in Table 2, and according to the previous results in Section 3.3, Section 3.4 and Section 3.5, the highest and lowest release rates and the content of the released OPO were observed in the NC9 and NC3 powders, respectively; because the formed WPC–pectin complexes in the NC3 sample were protecting the OPO more efficiently, and a lower release rate was observed. On the other hand, the formation of WPC–pectin complexes in the NC9 powder was weaker, so the release of OPO was higher.

The statistical analysis also revealed that the difference between the samples was significant (*p* < 0.05). In general, it can be concluded that, depending on the final food formulations proposed for these encapsulated powders and their conditions, each powder could deliver a good taste in the food products (based on the release data). At the same time, the remaining (unreleased) OPO could go into the gastrointestinal tract as a functional bioactive and be released there [32].

## 4. Conclusions

In this study, the release profile of OPO from the freeze-dried encapsulated powders containing WPC–pectin complexes was evaluated at different temperatures of 30, 60, and 90 °C at different pH values of 3, 7, and 11. At first, the EE% of OPO in the encapsulated powders was evaluated and obtained in a 70–88% range, and the size of the particles was determined by an AFM analysis. The results of the release profiles showed that the highest and lowest release rates in all the samples were in the alkaline (pH = 11) and the acidic (pH = 3) conditions, respectively. In addition, the results of the modeling for the release data indicated that Higuchi was the best-fitted experimental model for the release data, with a high correlation coefficient, and the best modeling was performed in alkaline conditions, based on the correlation coefficients for all three powders. The release of OPO in the simulated salivary system was also investigated, and the results showed that, within 2 min, about 50% of the OPO was released. The statistical analysis results also showed a significant difference (*p* < 0.05) between the samples; and the remaining content can also be released within the intestine.

## Figures and Tables

**Table 1:** Parameters of kinetics modeling for the release of orange peel oil from NC3 powder at different conditions.

	Zero-Order			First-Order		
Treatments	R^2^	K	RMSE	R^2^	K	RMSE
T = 30 °C, pH = 3	0.90 ± 0.01	0.04 ± 0.01	7.64 ± 0.05	0.83 ± 0.01	0.001 ± 0.000	0.02 ± 0.00
T = 30 °C, pH = 7	0.87 ± 0.02	0.08 ± 0.01	18.57 ± 0.08	0.84 ± 0.02	0.001 ± 0.000	0.05 ± 0.00
T = 30 °C, pH = 11	0.97 ± 0.01	0.11 ± 0.02	11.82 ± 0.05	0.99 ± 0.01	0.002 ± 0.000	0.02 ± 0.00
T = 60 °C, pH = 3	0.89 ± 0.02	0.05 ± 0.01	11.07 ± 0.09	0.75 ± 0.03	0.001 ± 0.000	0.04 ± 0.00
T = 60 °C, pH = 7	0.95 ± 0.01	0.09 ± 0.01	11.89 ± 0.05	0.94 ± 0.01	0.002 ± 0.000	0.03 ± 0.00
T = 60 °C, pH = 11	0.87 ± 0.02	0.10 ± 0.02	23.76 ± 0.10	0.93 ± 0.01	0.003 ± 0.000	0.07 ± 0.01
T = 90 °C, pH = 3	0.81 ± 0.03	0.06 ± 0.01	17.15 ± 0.11	0.33 ± 0.04	0.001 ± 0.000	0.09 ± 0.01
T = 90 °C, pH = 7	0.82 ± 0.01	0.08 ± 0.01	22.63 ± 0.09	0.65 ± 0.02	0.002 ± 0.000	0.10 ± 0.01
T = 90 °C, pH = 11	0.88 ± 0.01	0.10 ± 0.02	21.93 ± 0.20	0.92 ± 0.01	0.003 ± 0.000	0.08 ± 0.00
	**Higuchi**			**Hixson–Crowell**		
**Treatments**	**R^2^**	**K**	**RMSE**	**R^2^**	**K**	**RMSE**
T = 30 °C, pH = 3	0.96 ± 0.01	0.71 ± 0.10	1.92 ± 0.03	0.80 ± 0.01	0.000 ± 0.000	0.10 ± 0.00
T = 30 °C, pH = 7	0.96 ± 0.01	1.56 ± 0.21	6.30 ± 0.07	0.78 ± 0.01	0.001 ± 0.000	0.26 ± 0.01
T = 30 °C, pH = 11	0.94 ± 0.01	1.70 ± 0.28	6.30 ± 0.11	0.98 ± 0.01	0.001 ± 0.000	0.11 ± 0.00
T = 60 °C, pH = 3	0.97 ± 0.01	1.07 ± 0.16	2.27 ± 0.04	0.70 ± 0.02	0.001 ± 0.000	0.18 ± 0.01
T = 60 °C, pH = 7	0.99 ± 0.01	1.58 ± 0.26	2.35 ± 0.04	0.89 ± 0.01	0.001 ± 0.000	0.20 ± 0.01
T = 60 °C, pH = 11	0.96 ± 0.02	2.26 ± 0.18	4.79 ± 0.06	0.82 ± 0.01	0.002 ± 0.000	0.40 ± 0.01
T = 90 °C, pH = 3	0.83 ± 0.02	1.65 ± 0.32	6.27 ± 0.08	0.14 ± 0.02	0.001 ± 0.000	0.41 ± 0.01
T = 90 °C, pH = 7	0.88 ± 0.01	2.06 ± 0.04	6.99 ± 0.07	0.47 ± 0.02	0.002 ± 0.000	0.49 ± 0.02
T = 90 °C, pH = 11	0.94 ± 0.01	2.37 ± 0.06	5.97 ± 0.08	0.78 ± 0.03	0.002 ± 0.000	0.45 ± 0.02

**Table 2:** The release of orange peel oil from three WPC–pectin complex powders in the simulated salivary system.

	Release (%)	
Samples	1st Minute	2nd Minute
NC3	16.8	36.4
NC6	26.1	49.3
NC9	29.8	56.1

## Data Availability

The data are available from the corresponding author.

## References

[1] Ghasemi S., Jafari S., Assadpour E., Khomeiri M. (2017). Production of pectin-whey protein nano-complexes as carriers of orange peel oil. Carbohydr. Polym..

[2] Ghasemi S., Jafari S., Assadpour E., Khomeiri M. (2018). Nanoencapsulation of d-limonene within nanocarriers produced by pectin-whey protein complexes. Food Hydrocoll..

[3] Estrella-Osuna D.E., Tapia-Hernández J.A., Ruíz-Cruz S., Márquez-Ríos E., Ornelas-Paz J.D., Del-Toro-Sánchez C.L., Ocaño-Higuera V.M., Rodríguez-Félix F., Estrada-Alvarado M.I., Cira-Chávez L.A. (2022). Nanoencapsulation of Eggplant (*Solanum melongena* L.) Peel Extract in Electrospun Gelatin Nanofiber: Preparation, Characterization, and In Vitro Release. Nanomaterials.

[4] Jones O., Decker E., McClements D. (2010). Thermal analysis of β-lactoglobulin complexes with pectins or carrageenan for production of stable biopolymer particles. Food Hydrocoll..

[5] Jafari S., Assadpoor E., He Y., Bhandari B. (2008). Encapsulation efficiency of food flavours and oils during spray drying. Dry. Technol..

[6] Jafari S., Fathi M., Mandala I. (2015). Emerging product formation. Food Waste Recovery.

[7] Tapia-Hernández J.A., Rodríguez-Felix F., Juárez-Onofre J.E., Ruiz-Cruz S., Robles-García M.A., Borboa-Flores J., Wong-Corral F.J., Cinco-Moroyoqui F.J., Castro-Enríquez D.D., Del-Toro-Sánchez C.L. (2018). Zein-polysaccharide nanoparticles as matrices for antioxidant compounds: A strategy for prevention of chronic degenerative diseases. Food Res. Int..

[8] Assadpour E., Jafari S.M., Maghsoudlou Y. (2017). Evaluation of folic acid release from spray dried powder particles of pectin-whey protein nano-capsules. Int. J. Biol. Macromol..

[9] Mohnen D. (2008). Pectin structure and biosynthesis. Curr. Opin. Plant Biol..

[10] Fathi M., Mozafari M., Mohebbi M. (2012). Nanoencapsulation of food ingredients using lipid based delivery systems. Trends Food Sci. Technol..

[11] Fathi M., Varshosaz J., Mohebbi M., Shahidi F. (2013). Hesperetin-loaded solid lipid nanoparticles and nanostructure lipid carriers for food fortification: Preparation, characterization, and modeling. Food Bioprocess Technol..

[12] Jiménez-Alvarado R., Beristain C., Medina-Torres L., Román-Guerrero A., Vernon-Carter E. (2009). Ferrous bisglycinate content and release in W1/O/W2 multiple emulsions stabilized by protein–polysaccharide complexes. Food Hydrocoll..

[13] Hosseini S., Emam-Djomeh Z., Sabatino P., Van der Meeren P. (2015). Nanocomplexes arising from protein-polysaccharide electrostatic interaction as a promising carrier for nutraceutical compounds. Food Hydrocoll..

[14] Zimet P., Livney Y. (2009). Beta-lactoglobulin and its nanocomplexes with pectin as vehicles for ω-3 polyunsaturated fatty acids. Food Hydrocoll..

[15] Bagheri L., Madadlou A., Yarmand M., Mousavi M. (2013). Nanoencapsulation of date palm pit extract in whey protein particles generated via desolvation method. Food Res. Int..

[16] Jafari S., He Y., Bhandari B. (2007). Encapsulation of nanoparticles of d-limonene by spray drying: Role of emulsifiers and emulsifying techniques. Dry. Technol..

[17] Peinado I., Lesmes U., Andrés A., McClements J. (2010). Fabrication and morphological characterization of biopolymer particles formed by electrostatic complexation of heat treated lactoferrin and anionic polysaccharides. Langmuir.

[18] Matias R., Ribeiro P., Sarraguça M., Lopes J. (2014). A UV spectrophotometric method for the determination of folic acid in pharmaceutical tablets and dissolution tests. Anal. Methods.

[19] Dash S., Murthy P., Nath L., Chowdhury P. (2010). Kinetic modeling on drug release from controlled drug delivery systems. Acta Pol Pharm.

[20] Ades H., Kesselman E., Ungar Y., Shimoni E. (2012). Complexation with starch for encapsulation and controlled release of menthone and menthol. LWT-Food Sci. Technol..

[21] Amaechi B., Higham S. (2001). In vitro remineralisation of eroded enamel lesions by saliva. J. Dent..

[22] Chang Y., McClements D. (2014). Optimization of orange oil nanoemulsion formation by isothermal low-energy methods: Influence of the oil phase, surfactant, and temperature. J. Agric. Food Chem..

[23] Yamaguchi M., Kanemori T., Kanemaru M., Takai N., Mizuno Y., Yoshida H. (2004). Performance evaluation of salivary amylase activity monitor. Biosens. Bioelectron..

[24] Hosseini S., Zandi M., Rezaei M., Farahmandghavi F. (2013). Two-step method for encapsulation of oregano essential oil in chitosan nanoparticles: Preparation, characterization and in vitro release study. Carbohydr. Polym..

[25] Crank J. (1975). The Mathematics of Diffusion.

[26] Lutz R., Aserin A., Wicker L., Garti N. (2009). Release of electrolytes from W/O/W double emulsions stabilized by a soluble complex of modified pectin and whey protein isolate. Colloids Surf. B Biointerfaces.

[27] Mohammadi A., Jafari S., Assadpour E., Esfanjani A. (2016). Nano-encapsulation of olive leaf phenolic compounds through WPC–pectin complexes and evaluating their release rate. Int. J. Biol. Macromol..

[28] Lakkis J. (2016). Encapsulation and Controlled Release Technologies in Food Systems.

[29] Liu F., Avena-Bustillos R., Chiou B.-S., Li Y., Ma Y., Williams T., Wood D., McHugh T., Zhong F. (2017). Controlled-release of tea polyphenol from gelatin films incorporated with different ratios of free/nanoencapsulated tea polyphenols into fatty food simulants. Food Hydrocoll..

[30] Esfanjani A., Jafari S., Assadpoor E., Mohammadi A. (2015). Nano-encapsulation of saffron extract through double-layered multiple emulsions of pectin and whey protein concentrate. J. Food Eng..

[31] Mircioiu I., Anuta V., Ibrahim N., Mircioiu C. (2012). Dissolution of tamoxifen in biorelevant media. A two phase release model. Farmacia.

[32] Devi N., Sarmah M., Khatun B., Maji T.K. (2017). Encapsulation of active ingredients in polysaccharide–protein complex coacervates. Adv. Colloid Interface Sci..

